# The Relationship Between Symptom Change and Use of a Web-Based Self-Help Intervention for Parents of Children With Externalizing Behavior Disorders: Exploratory Study

**DOI:** 10.2196/54051

**Published:** 2024-09-04

**Authors:** Laura Wähnke, Christina Dose, Marie-Theres Klemp, Judith Mühlenmeister, Julia Plück, Manfred Döpfner

**Affiliations:** 1 School for Child and Adolescent Cognitive Behavior Therapy Faculty of Medicine and University Hospital Cologne The University of Cologne Cologne Germany; 2 Department of Child and Adolescent Psychiatry, Psychosomatics and Psychotherapy Faculty of Medicine and University Hospital Cologne The University of Cologne Cologne Germany

**Keywords:** web-based self-help, eHealth, parent management training, externalizing symptom, ADHD, attention-deficit hyperactivity disorder, self-help, use, child, children, parent, parents, management, management training, symptom, symptoms, caregiver, ODD, oppositional defiant disorder, treatment, web-based, caregivers, longitudinal data

## Abstract

**Background:**

Web-based self-help (WASH) has been found to be effective in the treatment of child externalizing behavior disorders. However, research on the associations of caregivers’ use of WASH and symptom changes of child externalizing behaviors is lacking.

**Objective:**

This study examined the longitudinal and reciprocal associations between the use of WASH by caregivers of children with externalizing behavior disorders and their children’s externalizing behavior symptoms.

**Methods:**

Longitudinal data of 276 families from 2 intervention conditions of a randomized controlled trial (either unguided or supported by a therapist over the phone) were analyzed. Caregiver- and clinician-rated child externalizing behavior symptoms were assessed before (T1), in the middle (T2), and after the 6-month WASH intervention (T3). Additionally, 2 indicators of the caregivers’ use of the WASH intervention were considered: number of log-ins (frequency) and the percentage of completed material (intensity). Associations of caregivers’ use during early (T1-T2) and late (T2-T3) treatment with child externalizing behavior symptoms were analyzed using path analyses (structural equation modeling).

**Results:**

Frequency and intensity of use were higher during the first 3 months than during the next 3 months of the intervention period. The number of log-ins at early treatment was significantly but weakly associated with caregiver-reported child externalizing behavior symptoms in the long term (T3). Moreover, caregiver-reported child externalizing severity at T2 predicted the number of log-ins in the late treatment. The results were not replicated when considering the percentage of completed material as a measure of use or when considering clinician ratings of child externalizing behavior symptoms.

**Conclusions:**

The findings provide the first, albeit weak, evidence for longitudinal associations between caregivers’ use of WASH and improvements in caregiver-rated child externalizing behavior symptoms. However, as the associations were rather weak and could not be replicated across different rater perspectives and operationalizations of use, further research is needed to better understand these relations and their interplay with other putative influence factors (eg, quality of the implementation of the interventions, changes in parenting behaviors).

**Trial Registration:**

German Clinical Trials Register DRKS00013456; https://www.drks.de/DRKS00013456

**International Registered Report Identifier (IRRID):**

RR2-10.1186/s12888-020-2481-0

## Introduction

Parenting interventions have been shown to reduce oppositional defiant behavior problems in children [1,2]. Behavioral parent management training (PMT) has led to a reduced number of problematic situations of caregiver-child interactions reported by caregivers of children with attention-deficit hyperactivity disorder (ADHD) [3]. Although previous research has demonstrated both self-directed and face-to-face PMT to be effective in reducing child externalizing behavior disorders, outcome ratings of PMT vary across different assessors (eg, parents, clinicians, objective observations), with stronger evidence for PMT in caregiver reports [4,5]. Improvements have been reported by caregivers (completers) for conduct problems (*P*=.001) and hyperactivity symptoms (*P*<.001) [6]. Moreover, it remains unclear what drives symptom-related improvements: Although some studies indicate that parental attendance and engagement in face-to-face PMT are associated with greater symptom reduction for disruptive behavior, ADHD, and oppositional defiant disorder (ODD) symptoms [7], others indicate no differences in child behavior outcomes between mothers who complete versus mothers who drop out of PMT [6]. In a systematic review of preventive child mental health programs, higher levels of caregiver participation engagement (CPE) were associated with greater improvements in child internalizing and externalizing behavior symptoms [8]. Additionally, parental outcomes (eg, warm interactions, reduced physical punishment) seem to be associated with the quality of participation (rated by a therapist, eg, completion of between-session homework or the amount of participation in the group) rather than with mere attendance in PMT for conduct problems [9].

Web-based PMT is an easily accessible treatment alternative to face-to-face PMT, with proven effectiveness in the treatment of, for example, externalizing behavior disorders [10,11] and anxiety disorders [12]. There is evidence for the efficacy of web-based PMT regarding the reduction in conduct disorder and ADHD symptoms, with mostly small-to-moderate effect sizes [13-18]. However, self-help interventions often fail to keep caregivers engaged, rendering them difficult to complete [19,20]. In a 3-arm randomized controlled trial (RCT), we demonstrated that web-assisted self-help (WASH) combined with therapist telephone support (Döpfner et al, unpublished data, August 2024) is superior to routine clinical care, as well as WASH alone, in reducing clinician-rated child externalizing behavior symptoms [21]. Compared to face-to-face therapy and in line with other research on online interventions, our study revealed a relatively low intensity of use (average 35%), although the majority of participants (89.4%) logged in to the intervention at least once [22].

Considering self-directed interventions based on booklets for caregivers of children with externalizing behaviors (eg, booklets), parental adherence is associated with improved child externalizing behaviors [23]. Research on attrition and usage provides indications that more frequent users of eHealth report a decline in their perceived burden compared with an increased perceived burden reported by nonusers [24]. Regarding web-based PMT for caregivers of children with anxiety and depressive symptoms, parental engagement (defined as stronger orientation toward recommended use, ie, task completion) predicted (caregiver-rated) preventive parenting and lower impairment in the child’s quality of life [25]. However, parental engagement did not predict changes in (caregiver- and child-reported) internalizing symptoms. By contrast, the self-reported frequency of practicing skills (during the “Cool Little Kids” online program) was associated with a greater decrease in child anxiety symptoms [12].

Conversely, children’s severity of externalizing behaviors at baseline for the intervention on which this research is based has previously been found to be 1 of the predictors for the use of WASH [22]. There is evidence that parental perception of the severity of a child’s symptoms is predictive of their help-seeking behaviors [26]. However, factors that are associated with parental engagement in children’s mental health treatment have yielded divergent results, with some research indicating, for example, child mental health symptoms as a predictor for parental engagement and others not [8,27].

Clearly, the actual use of web-based interventions is a prerequisite for their efficacy. To date, there is no common sense of how the use of web-based interventions is conceptualized and operationalized, with measures ranging from direct measures (ie, self-report) to objective measures (ie, automatic data tracking of, eg, the number of log-ins) [28]. In the context of face-to-face treatment, the terms “engagement,” “participation,” and “adherence” are often used interchangeably; however, they include different therapy-related behaviors, from active participation during a session to practice implementation between sessions (eg, practically adapting parenting behaviors) [27]. In a systematic review, the most commonly reported measure for adherence is the number of log-ins to e-therapies [29]. However, a unidimensional operationalizing of the term’s use has been criticized by researchers [30,31]; for example, one can often log in to the intervention (frequency), while making little progress (intensity) in the intervention. These parameters, though, seem to be associated with one another [22,27].

To the best of our knowledge, no study on web-based PMT for child externalizing behaviors has analyzed the relationships between parental use and changes in child externalizing behavior symptoms. Moreover, associations between the use of online interventions and symptom changes have rarely been examined in other child mental health conditions. The few available studies differ regarding the type of intervention (eg, preventive program, booklet self-help, face-to-face group PMT), the target group (affected individuals, mostly adults, vs parents), operationalization of treatment use, outcome measures, and how outcome measures are assessed. In behavioral face-to-face PMT, research on the association between attendance and child externalizing symptom severity has yielded divergent results [8,27].

This study took an exploratory approach to examine the longitudinal and reciprocal associations between the use of WASH and child externalizing symptom severity using 3 subsequent assessment points of child externalizing behavior symptoms and in-between assessments of WASH uses. Although it might seem self-evident that the actual use of an intervention is a prerequisite for it to affect child externalizing behavior symptoms, previous research has yielded mixed findings in this regard [8,27]. Thus, we did not formulate specific a priori hypotheses for possible associations. Moreover, as a previous study using baseline data of the same data set as this study demonstrated the predictive value of baseline symptom severity for the subsequent use of WASH [22], we also exploratively examined longitudinal associations between previous symptom severity and subsequent use. As the agreement of different raters on child externalizing symptom severity is typically only low to moderate [4,5], we considered both caregiver and clinician ratings of child externalizing behavior symptoms to obtain a more comprehensive impression of their associations with use. Moreover, as previous research has often been criticized for considering only 1 possible measure of use, we considered both the number of log-ins (frequency of use) and the percentage of completed materials (intensity of use) to operationalize use. However, we chose to consider the number of log-ins for our primary analyses, as this is the measure most often used in previous research [29]. The study was based on the use of WASH PMT in terms of automatically tracked objective measures (number of log-ins, percentage of processed content) of using an online treatment, in which caregivers are free to take an interest-based approach in processing the training [22]. Findings for associations between the percentage of completed materials and child externalizing behavior symptoms are presented in a supplementary manner. The ultimate aim is to provide a basis for improving internet-delivered interventions, in turn contributing to the further development of an effective therapeutic supply for children with externalizing behavior disorders.

## Methods

### Study Design

Data for the analyses were collected as part of an effectiveness study on WASH [21]. The research compared 3 study conditions: (1) WASH alone, (2) WASH plus telephone-based support (WASH+SUPPORT), and (3) treatment as usual (TAU). The analyses included data from the first 2 conditions only. There were no restrictions regarding the use of further treatment options during study participation.

The RCT from which data for the analyses were gathered was registered at the German Clinical Trials Register (identifier: DRKS00013456; registered on January 3, 2018).

### Ethical Considerations

This study was approved by the Ethics Committee of the Medical Faculty of the University Hospital Cologne (Germany; approval number: 17-273) and was performed in accordance with the ethical standards laid down in the 1964 Declaration of Helsinki and its later amendments. All participating caregivers provided written informed consent before randomization.

### Participants

**Figure 1:**
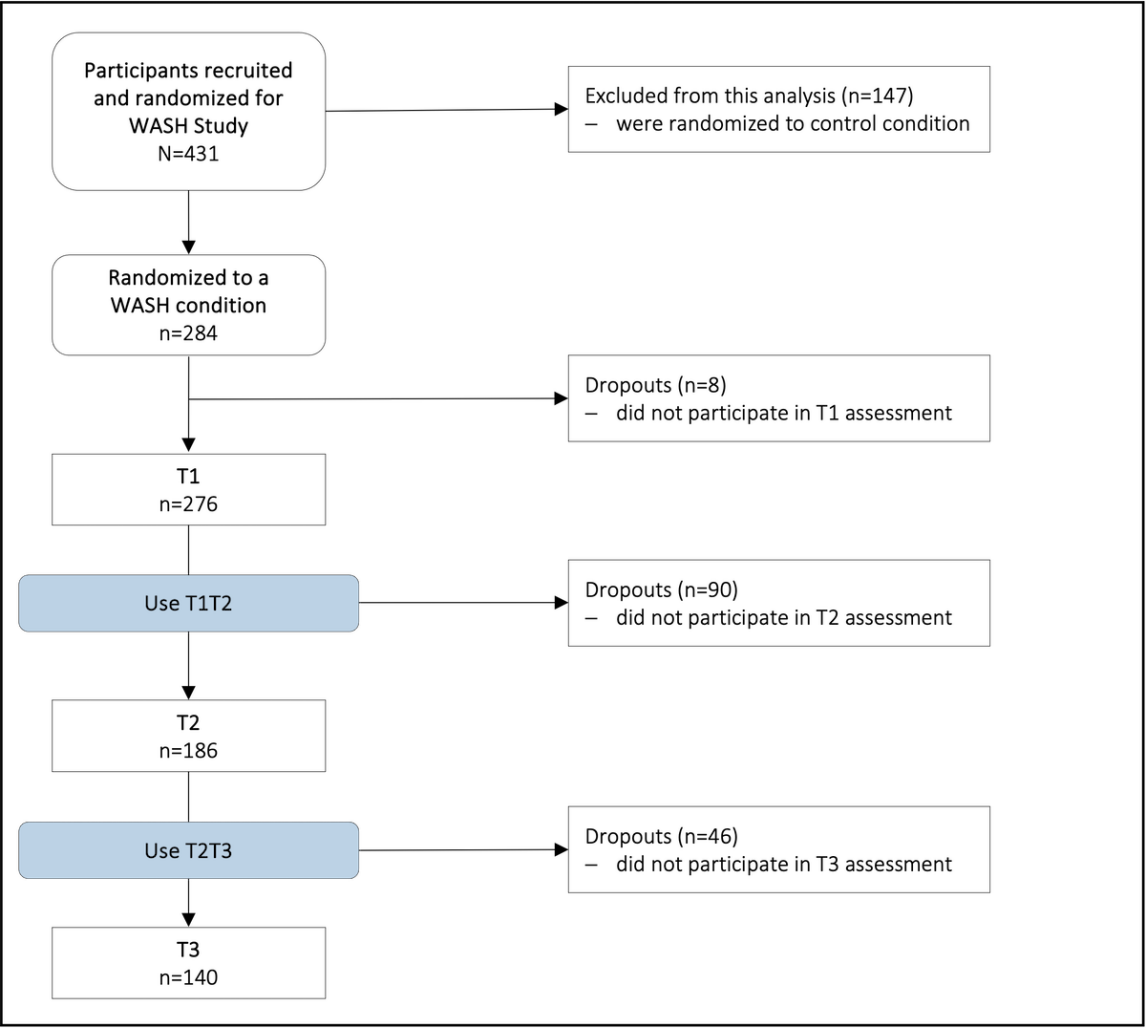
Participant flow. Note. T1: before the intervention; T2: in the middle of the intervention; T3: after the 6-month intervention; WASH: web-based self-help.

Participants were caregivers of children with externalizing behavior symptoms. The inclusion criteria were child age between 6 and 12 years and elevated levels of ADHD or ODD symptoms at the first assessment point (clinician rated during the caregiver interview). A diagnosis of mental retardation or autism spectrum disorder or an indication for inpatient treatment led to exclusion from the study. For subsequent analyses, we used a subsample of 276 caregivers who were randomized to the 2 intervention conditions: WASH (n=135, 48.9%) and WASH+SUPPORT (n=141, 51.1%); see Figure 1. Participating caregivers (n=147) in the control condition (TAU) were excluded from the analyses as they did not use the intervention, and thus, we could not assess use in this group.

### Intervention

The online intervention was based on previous manual-based self-help programs that have proven effective in reducing children’s ADHD or ODD symptoms [32-34]. The WASH intervention comprises 4 modules: solving behavioral problems, positive relationship with your child, self-care, and psychoeducation. We provided recommendations regarding usage, but caregivers were generally free to navigate the program according to their interests. Participants in the WASH+SUPPORT group received up to 6 support calls from a trained and supervised professional. A detailed description is provided in the published study protocol [21]. Participants in both study conditions received reminders if they had not logged in within 5 days after randomization. Users in the WASH group were then free to use the program and were contacted for assessment (12 weeks, T2; 24 weeks, T3 after T1). Although there was a significant overall intervention effect on child externalizing behaviors, as rated by a blinded clinician (Döpfner et al, unpublished data, August 2024), the superiority of the WASH+SUPPORT condition over the WASH condition, revealed by subsequent pairwise comparisons, was compensated in this analysis by using the condition as a covariate.

### Measures

Data were collected using a semistructured, telephone-based caregiver interview by a trained clinician and caregiver-rated online questionnaires before the intervention (T1), at a 3-month interim assessment point during the intervention (T2), and after the intervention (ie, at 6 months, T3).

#### Child Externalizing Behavior Symptoms

At each assessment point, a clinician rated the child’s externalizing behavior symptoms based on a semistructured interview with the participating caregiver (“Diagnostic Checklist for Externalizing Behavior Disorders,” DCL-EXT), conducted over the phone [35]. The interview covered ADHD symptoms (18 items) and ODD symptoms (8 items) according to the *International Classification of Diseases, 10th Revision* (*ICD-10*) and the *Diagnostic and Statistical Manual of Mental Disorders, Fifth Edition* (DSM-5) and has been proven to be a high-quality diagnostic instrument for externalizing behavior disorders [36]. For our analyses, an overall externalizing symptom score was calculated by averaging all item scores. In the study sample, the internal consistency of this score was satisfactory (Cronbach α=.79).

Corresponding to the clinician ratings, at each assessment point, caregivers rated their children’s externalizing behavior symptoms using 18 items of the German Symptom Checklist for ADHD and 8 items of the ODD subscale of the Symptom Checklist for Disruptive Behavior Disorders (“Symptom Checklist for Externalizing Behavior Disorders,” SCL-EXT) [35]. The items were answered on a 4-point Likert scale (ranging from 0 for *not at all* to 3 for *very much/particularly severe*) [35]. Again, the total externalizing symptom score (SCL-EXT) was computed by averaging all item scores. The instruments have demonstrated factorial validity and satisfactory internal consistency [35,37,38]. The internal consistency in the study sample was satisfactory (Cronbach α=.90) for the combined total SCL- EXT score.

#### Intervention Use

For each caregiver, an automatically generated log file was extracted 3 months after baseline (at T2) and 6 months after baseline (at T3), including the number of log-ins (ie, frequency of use) in the first 3 months (T1-T2) and the next 3 months (T2-T3), respectively [39,40]. Beyond that, we calculated the percentage of completed tasks/videos (ie, intensity of use, %) between T1 and T2 or between T2 and T3 for each participant by dividing the number of finished tasks and videos in a module/for a specific situation by the maximum number of tasks and videos provided in that module [22]. Reliability analyses for this processing progress scale yielded an acceptable internal consistency (Cronbach α=.78).

### Statistical Analysis

**Figure 2:**
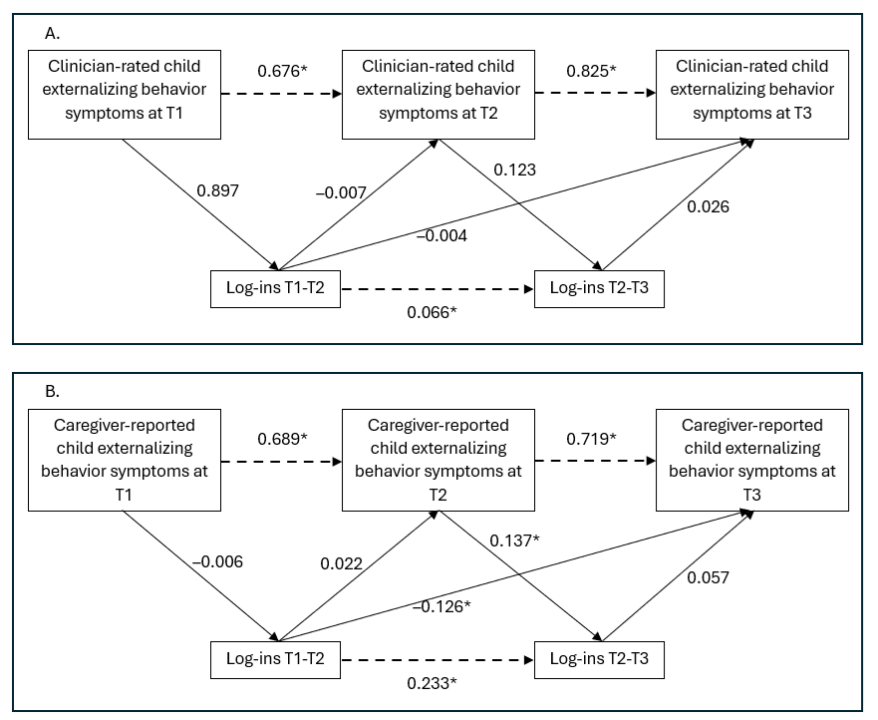
Results of the path models examining the association between the caregiver’s number of log-ins to the WASH intervention and clinician-rated (A) or caregiver-rated (B) child externalizing behavior symptoms. Note. Log-ins T1-T2: number of log-ins during the early intervention phase (months 0-3); log-ins T2-T3: number of log-ins during the late intervention phase (months 3-6); Tx: measuring time; dashed line indicates autoregressive directional correlations; *P<.05.

Analyses were conducted on the sample with complete questionnaires for at least T1. Before the main analyses, which included data from participants in both study conditions, independent samples *t* tests were performed to test for differences in child externalizing behavior symptoms between the study conditions at T1. To examine whether child externalizing behavior symptoms affect caregivers’ use of the WASH intervention and vice versa, we performed path analyses. In these analyses, we considered associations between the severity of child externalizing behavior symptoms at T1 and the caregivers’ use of the WASH intervention in the early intervention period (between T1 and T2), as well as associations between caregivers’ use in the early intervention period and child externalizing symptom severity at T2. Accordingly, we regarded the associations between symptom severity at T2 and caregivers’ use of the program during the late intervention period (between T2 and T3), as well as associations between this use in the late intervention period and symptom severity at T3. In addition to these paths, to account for temporal stability, we considered autoregressive correlations between child externalizing behavior symptoms measured at the different assessment points and between the use parameters assessed between the assessment points (see Figure 2).

The use of WASH took place between the measurements of children’s externalizing behavior symptoms. For our main analyses, we examined 2 different models, with externalizing behavior symptoms rated by either clinicians (see Figure 2A) or caregivers (see Figure 2B) and with caregivers’ frequency of use of WASH operationalized by the number of log-ins (log-ins in months 0-3 and log-ins in months 3-6)*.* We chose this measure of use for our primary analyses as it has been reported in most of the previous research on use-symptom association and, thus, allows for comparability with previous findings. Moreover, due to automatic data tracking, this measure seems reliable. However, we additionally conducted analogous analyses on the question of whether the results can be replicated when using a different operationalization of use (ie, percentage of completed materials; intensity of use). The findings for these additional analyses are presented in the online supplement for this paper. In all analyses, we controlled for the effect of the study condition (WASH and WASH+SUPPORT) on the use parameters and on symptoms.

We reported standardized parameter estimates (ß). To evaluate the model fit, we considered the comparative fit index (CFI) and the standardized root mean square residual (SRMR), in addition to *χ*^2^. In line with current recommendations, we considered CFI>0.90 and SRMR<0.08 as acceptable [22,41]. Despite its frequent use, we refrained from relying on the root mean square error of approximation (RMSEA), as this index is not suitable in the case of low degrees of freedom [42]. The analyses were conducted using the Statistical Package for the Social Sciences (SPSS) version 27 (IBM Corp) for descriptive statistics and *t* tests and Mplus version 7.4 (Muthén & Muthén) for path analyses.

## Results

### Sample Description

Table 1 summarizes the demographic and clinical characteristics of the participating caregivers and their children. At T1, on average, clinicians indicated elevated levels of child externalizing behavior symptoms (DCL-EXT: mean 1.52, SD 0.36). Likewise, caregivers reported clinically relevant child externalizing behavior symptoms (SCL-EXT: mean 1.70, SD 0.46). The independent samples *t* tests revealed no significant differences between the 2 intervention conditions in caregiver-reported child externalizing behavior symptoms at baseline (t_282_=1.32, *P*=.19). After 6 months (DCL-EXT T3: mean 1.08, SD 0.44; SCL-EXT T3: mean 1.36, SD 0.50), symptom levels were still considered as elevated, according to orienting evaluation without normative standards [35]. However, both clinician-rated (t_193_=16.33, *P*<.001) and caregiver-rated (t_158_=10.70, *P*<.001) child externalizing behavior symptoms declined significantly between T1 and T3.

On average, caregivers logged in to the WASH intervention 5 times (SD 4.38, range 0-18) during the early intervention period (months 0-3), with significantly fewer log-ins (mean 0.53, SD 1.20, range 0-9) during the late intervention period (months 3-6; t_275_=17.40, *P*<.001). Additionally, the percentage of completed material was significantly higher in the first intervention period (mean 31.88, SD 26.08, range 0-96.70) than in the second intervention period (mean 1.93, SD 5.83, range 0-31.20; t_275_=18.76, *P*<.001).

**Table 1:** Demographic information about caregivers and children (N=276).

Variable		Value
**Caregivers**		
	Sex (women), n (%)	254 (92.0)
	Age (years), mean (SD, range)^a^	42.93 (5.95, 26.61-1.54)
**Children**		
	Sex (boys), n (%)	226 (81.9)
	Age (years), mean (SD, range)	9.35 (1.73, 6.00-12.97)
***ICD-10*^b^ diagnosis by local health care provider, n (%)**		
	Suspected ADHD^c^	64 (23.2)
	F90.0 Hyperkinetic Disorders, Disturbance of Activity and Attention	160 (58.0)
	F90.1 Hyperkinetic Conduct Disorder	42 (15.2)
	F90.8 Other Hyperkinetic Disorders or Hyperkinetic Disorder and F90.9 Hyperkinetic Disorders or Hyperkinetic Disorder, Unspecified	4 (1.4)
	F98.8 Attention-Deficit Disorder without Hyperactivity	6 (2.2)

^a^Reduced N=253 due to 1 missing value at baseline.

^b^*ICD-10*: *International Classification of Diseases, 10th Revision*.

^c^ADHD: attention-deficit hyperactivity disorder.

### Associations of Caregivers’ Use With Symptoms of Children

Results of the path analyses on the associations between the number of log-ins and changes in child externalizing symptom severity are reported in Figure 2 and Table S1 in Multimedia Appendix 1. The model fit was acceptable for both models (associations between clinician-rated child externalizing behavior symptoms and the number of log-ins: CFI=0.97, SRMR=0.03; associations between caregiver-rated child externalizing behavior symptoms and the number of log-ins: CFI=0.92, SRMR=0.04). Although significant, the *χ*^2^ value depends strongly on the degrees of freedom, which, at *df*=2, were considered acceptable (see Table S2 in Multimedia Appendix 1) [43,44]. The primary analyses yielded no significant associations between the number of log-ins and clinician-rated child externalizing behavior symptoms (see Figure 2 and Table S1 in Multimedia Appendix 1). A small significant negative association was found between the number of log-ins (months 0-3) and the caregiver-reported child externalizing behavior symptoms in the long term (T3; ß=–.13, *P*=.29). Moreover, the caregiver-reported severity of child externalizing behavior symptoms at T2 was significantly associated with a higher number of log-ins in the later phase (ß=.14, *P*=.29). The significant results must be classified as small effects based on the standardized ß coefficient [45]. To examine whether the findings for the use-symptom associations may be replicated when using a different operationalization of use, we conducted secondary analyses according to the main analyses but applied the percentage of completed materials as a measure of use (see Table S3 in Multimedia Appendix 1). The model fit and *χ*^2^ for both secondary models were acceptable, too (see Table S2 in Multimedia Appendix 1). No significant associations were found, neither when considering clinician ratings nor when regarding caregiver ratings of child externalizing behavior symptoms.

## Discussion

### Principal Findings

To the best of our knowledge, this study is the first to systematically investigate the relationship between caregivers’ use of an online intervention and changes in their children’s externalizing behavior symptoms. Overall, use was low, especially during the second half of the intervention period. Our results hint at a bidirectional, albeit small, association between the caregiver’s frequency of use (ie, number of log-ins) and changes in caregiver-reported child externalizing behavior symptoms. That is, first, the more log-ins during the early phase, the less severe the externalizing behavior symptoms reported by caregivers in the longer term. Second, in contrast, the more severe the caregiver-rated externalizing behavior symptoms, the more frequent the log-ins to the intervention in the subsequent late intervention phase (months 3-6). However, none of the other use-symptom associations in this model were significant. Moreover, we were not able to replicate these findings when we considered clinician-rated rather than caregiver-rated externalizing behavior symptoms, nor when we operationalized caregivers’ use by the percentage of completed materials (intensity of use).

Consistent with research on the use of online treatment for depression, we found both more log-ins and higher task completion rates (frequency and intensity) in the first than in the second half of the intervention [46]. For the second intervention phase (months 3-6), the mean overall number of log-ins was low. Furthermore, the overall progress during the 6-month treatment was relatively low, with only about one-third of the program being processed, on average. In view of previous research, it is clear that low completion rates are a general problem of internet-delivered interventions [20,47]. In fact, participants did not receive clear guidelines regarding use but were allowed to work on the intervention and content according to their interests, and full program completion was neither recommended nor necessary, since the program offers a wide range of options for usage and parents are asked to choose the components that best suit their needs. As previous analyses of the data used in this study revealed that personal telephone contact is a main predictor of enhanced use [22], the lack of counseling support calls (during months 3-6) might explain the significantly lower use in this period. The lower use in this period, which was additionally associated with lower variance (see Table S4 in Multimedia Appendix 1), may contribute to the explanation of the small effect sizes and the nonsignificant associations in the models, including clinician-rated child externalizing behavior symptoms or the percentage of completed materials as a measure of use.

Considering the findings of at least some associations between caregivers’ use of the intervention and child externalizing behavior symptoms, the low use underlines the need for measures to foster engagement in online PMT to improve intervention outcomes. Previous research has demonstrated that use can be enhanced by some kind of support (eg, personal contact over the phone or chat functions, reminders) [22,48]. Contrary to previous findings of no significant use-symptom associations for PMT in the field of child anxiety disorders [25], our results hint at some longitudinal associations between the number of log-ins to the WASH intervention and caregiver-reported child externalizing behavior symptoms.

Notably, we not only found that (1) single aspects (frequency) of parental use of WASH are associated with externalizing behavior symptoms in the longer term but also observed that (2) externalizing symptom severity during treatment predicts later frequency of use. Although the effects were rather weak, and findings varied for different (but correlated) operationalizations of use (r=0.73, *P*≤.001) [22], we consider these results as providing initial exploratory evidence for use-symptom associations. The different results for frequency and intensity of use underline the need for a differentiated consideration of these 2 parameters [30], as they capture 2 different facets of use. Although the number of log-ins merely reflects participation in the program, the percentage of use provides an indication of the depth of processing of the program content. Based on the available information from the study, we cannot conclude why significant associations with symptoms were found for the number of log-ins and not for the percentage of use. Maybe the different findings might be explained by influences of child characteristics, disorder characteristics (eg, symptom severity), or caregiver characteristics (ie, own inattention problems), which might be related to either use behavior and child externalizing symptom severity or both. However, further research is needed first to determine whether the results of this study may be replicated and then to examine further reasons for the differing results for the number of log-ins and the percentage of use.

The negative association between frequency of use and subsequent symptom severity could not be replicated when regarding clinician-rated child externalizing behavior symptoms. Previous research on PMT aiming to compare/validate different outcome measures across different assessors (caregiver, teacher, clinician) has likewise found a lack of congruence across different raters [5]. The authors concluded that caregivers may overestimate the effects of PMT, potentially due to the resources they have invested in treatment use (effort justification). Moreover, changes in caregivers’ perceptions of children’s externalizing behavior symptoms lead to greater tolerance, leading them to rate behavioral problems as less severe [22]. Future research is needed to replicate our findings.

Overall, despite significant findings on some variables, the relationship between frequency of use and change in caregiver-rated child externalizing behavior symptoms is not strong. Thus, we may conclude that simply improving the use of the WASH program is insufficient to enhance treatment outcomes in clinical practice. Other factors that were not controlled for in these analyses may be more important for explaining the differences in outcomes and might be a more favorable starting point for improving interventions (eg, emotional and behavioral problems and competencies of the parents, quality of intervention implementation). For example, internet-delivered PMT—from a theoretical and practical perspective—seems to affect parenting skills or parental psychopathology [12,25], and previous research has demonstrated that the effects of PMT on child externalizing behavior symptoms are mediated by a change in (mainly negative) parenting behaviors [49]. A deeper examination of such additional factors and their interplay is necessary to obtain a more comprehensive impression of the processes leading to symptom changes and to draw conclusions on how to improve treatment outcomes in clinical practice. It is conceivable that the use of web-based PMT is a prerequisite for change in both child- and parent-related variables and that there is a complex interplay between these variables.

Future studies should examine more complex models, including additional moderators and mediators of the effects of web-based PMT on child externalizing behavior symptoms (eg, parental skills practice [12], parenting behaviors), and additionally focus on the use of individual techniques (eg, stimulus control, contingency management) to gain a deeper understanding of the relative contribution of treatment use and of the particular mechanisms that lead to symptom improvements. A combination of objective and subjective measures of the individual model components should be used to increase the validity of the findings. Moreover, measures to enhance treatment use (eg, additional support calls) and their relative importance for enhancing treatment outcomes should be examined in more detail. Recently, microtrials have examined the effectiveness of specific elements of face-to-face PMT (eg, stimulus control techniques vs contingency management) [50,51]. Transferring this approach to web-based PMT and linking it to the measures of treatment use may help further study and explain the associations between use parameters and symptom changes.

### Strengths and Limitations

Although the study sample is larger than in many other studies, it is nevertheless small for this type of analysis, limiting the possibility of detecting significant associations [52]. Moreover, several analyses were performed, increasing the risk of detecting significant effects by chance. Unfortunately, no measures on the practice and implementation (homework practice) of, for example, problem-solving strategies and contingency management into daily life were conducted. Moreover, we cannot guarantee that users engaged with the content rather than merely absolving the intervention, as we did not conduct knowledge quizzes to prove the CPE. The use variables are objective measures extracted from the program but do not indicate whether a caregiver changed parenting behaviors following treatment. Future research should include these variables (CPE in sessions and between sessions and parenting behaviors) to examine their contribution to symptom changes connected with caregivers’ use of WASH.

The strengths of the study are that we used 2 objective measures of use metrics (log data) and assessed child externalizing behavior symptoms from 2 different perspectives (ie, caregivers and clinicians).

### Conclusion

The analyses in this study provide some, albeit limited, support for the directional, longitudinal associations of (1) the caregiver’s early number of log-ins to WASH with child externalizing behavior symptoms in the longer term and (2) the severity of child externalizing behavior symptoms as an immediate predictor of following frequency of log-ins during the late intervention period. Although the results were inconsistent across different operationalizations of treatment use and raters (clinician vs caregiver) and although the effects were rather weak, these analyses provide the first evidence for use-symptom associations in web-based PMT for child externalizing behavior problems. Future research could try to replicate the results and consider complex models, including mediators and moderators of treatment outcomes (eg, parenting behaviors, parental psychopathology, sociodemographic variables, and effective elements). Ultimately, the respective results could be used to develop measures to improve the use of (web-based) PMT in clinical practice to enhance treatment outcomes.

## Appendix

Multimedia Appendix 1 Parameters for main analyses, model fit for all path models calculated on the association of use with the child’s overall externalizing symptoms, parameters for secondary analyses, and description of usage parameters (early and late).

## Abbreviations

ADHD: attention-deficit hyperactivity disorder
CFI: comparative fit index
CPE: caregiver participation engagement
DCL-EXT: Diagnostic Checklist for Externalizing Behavior Disorders
*ICD-10*: *International Classification of Diseases, 10th Revision*
ODD: oppositional defiant disorder
PMT: parent management training
RCT: randomized controlled trial
SCL-EXT: Symptom Checklist for Externalizing Behavior Disorders
SRMR: standardized root mean square residual
TAU: treatment as usual
WASH: web-based self-help

## Data Availability

The data sets used and analyzed during the study are available from the corresponding author upon reasonable request.
