# Supplementary material for: The Relationship Between Symptom Change and Use of a Web-Based Self-Help Intervention for Parents of Children With Externalizing Behavior Disorders: Exploratory Study
Source: JMIR Pediatr Parent. 2024 Sep 4;7:e54051. doi: 10.2196/54051 (PMC11411226; doi:10.2196/54051)
Supplement: Multimedia Appendix 1 [file pediatrics_v7i1e54051_app1.docx]

**Table S1.** Parameters for main analyses: associations of child externalizing symptoms (clinician- and caregiver-rated) and number of logins to the intervention

| Model | Path | *ß* | *SE* | *p* |
| --- | --- | --- | --- | --- |
|  | | | | |
| A: Clinician-rated | | | | |
|  | Child externalizing symptoms at T1 🡪 child externalizing symptoms at T2 | 0.62* | 0.04 | < .001 |
|  | Child externalizing symptoms at T2 🡪 child externalizing symptoms at T3 | 0.76* | 0.03 | < .001 |
|  | Logins T1-T2 🡪 logins T2-T3 | 0.24* | 0.06 | < .001 |
|  | Child externalizing symptoms at T1 🡪 logins T1-T2 | 0.08 | 0.06 | .176 |
|  | Logins T1-T2 🡪 child externalizing symptoms at T2 | -0.07 | 0.05 | .158 |
|  | Logins T1-T2 🡪 child externalizing symptoms at T3 | -0.04 | 0.05 | .437 |
|  | Child externalizing symptoms at T2 🡪 logins T2-T3 | 0.04 | 0.06 | .467 |
|  | Logins T2-T3 🡪 child externalizing symptoms at T3 | 0.07 | 0.04 | .101 |
|  | | | | |
| B: Caregiver-rated | | | | |
|  | Child externalizing symptoms at T1 🡪 child externalizing symptoms at T2 | 0.69* | 0.04 | < .001 |
|  | Child externalizing symptoms at T2 🡪 child externalizing symptoms at T3 | 0.72* | 0.04 | < .001 |
|  | Logins T1-T2 🡪 logins T2-T3 | 0.23* | 0.06 | < .001 |
|  | Child externalizing symptoms at T1 🡪 logins T1-T2 | -0.01 | 0.06 | .919 |
|  | Logins T1-T2 🡪 child externalizing symptoms at T2 | 0.02 | 0.06 | .687 |
|  | Logins T1-T2 🡪 child externalizing symptoms at T3 | -0.13* | 0.06 | .029 |
|  | Child externalizing symptoms at T2 🡪 logins T2-T3 | 0.14* | 0.06 | .029 |
|  | Logins T2-T3 🡪 child externalizing symptoms at T3 | 0.06 | 0.05 | .157 |

Note: A-B= model reference according to Figure 2 and with reference to Table S2, T1=pre-treatment (baseline), T2= 3 months after baseline, T3= 6 months after baseline; T1-T2= number of logins months 0 to 3, T2-T3= number of logins months 3 to 6; *p<.05.

**Table S2.** Model fit for all path models calculated on the association of use with the child’s overall externalizing symptoms

| Model & Path | χ^2^ | | | CFI | SRMR |
| --- | --- | --- | --- | --- | --- |
|  | χ^2^ | *df* | *p* |  |  |
|  |  |  |  |  |  |
| A: Clinician-rated child externalizing symptoms  and number of logins | 11.81 | 2 | 0.00 | 0.97 | 0.026 |
| B: Caregiver-rated child externalizing symptoms and number of logins | 24.30 | 2 | 0.00 | 0.92 | 0.038 |
|  |  |  |  |  |  |
| a: Clinician-rated child externalizing symptoms  and percentage of completed tasks/videos (%) | 15.17 | 2 | 0.00 | 0.96 | 0.030 |
| b: Caregiver-rated child externalizing symptoms and percentage of completed tasks/videos (%) | 25.63 | 2 | 0.00 | 0.90 | 0.039 |
|  |  |  |  |  |  |

Note: A-B= model reference according to Figure 2 and Table S1, a-b= model reference according to Table S3; *p<.05.

**Table S3.** Parameters for secondary analyses: associations of child externalizing symptoms (clinician- and caregiver-rated) and the percentage of completed material (%)

| Model | Path | | *ß* | *SE* | *p* |
| --- | --- | --- | --- | --- | --- |
|  | | | | | |
| a: Clinician-rated | | | | | |
|  | Child externalizing symptoms at T1 🡪 child externalizing symptoms at T2 | | 0.63* | 0.04 | < .001 |
|  | Child externalizing symptoms at T2 🡪 child externalizing symptoms at T3 | | 0.76* | 0.03 | < .001 |
|  | Completed material T1-T2 🡪 completed material T2-T3 | | 0.05 | 0.06 | .405 |
|  | Child externalizing symptoms at 🡪 completed material T1-T2 | | 0.08 | 0.06 | .181 |
|  | Completed material T1-T2 🡪 child externalizing symptoms at T2 | | -0.09 | 0.05 | .084 |
|  | Completed material T1-T2 🡪 child externalizing symptoms at T3 | | -0.01 | 0.05 | .843 |
|  | Child externalizing symptoms at T2 🡪 completed material T2-T3 | | -0.04 | 0.06 | .056 |
|  | Completed material T2-T3 🡪 child externalizing symptoms at T3 | | 0.01 | 0.04 | .889 |
|  | | | | | |
| b: Caregiver-rated | | | | | |
|  | Child externalizing symptoms at T1 🡪 child externalizing symptoms at T2 | | 0.68* | 0.04 | < .001 |
|  | Child externalizing symptoms at T2 🡪 child externalizing symptoms at T3 | | 0.46* | 0.07 | < .001 |
|  | Completed material T1-T2 🡪 completed material T2-T3 | | 0.04 | 0.06 | .519 |
|  | Child externalizing symptoms at T1 🡪 completed material T1-T2 | | 0.03 | 0.06 | .574 |
|  | Completed material T1-T2 🡪 child externalizing symptoms at T2 | | -0.09 | 0.06 | .104 |
|  | Completed material T1-T2 🡪 child externalizing symptoms at T3 | | 0.01 | 0.06 | .828 |
|  | Child externalizing symptoms at T2 🡪 completed material T2-T3 | | -0.12 | 0.09 | .202 |
|  | Completed material T2-T3 🡪 child externalizing symptoms at T3 | | -0.05 | 0.05 | .249 |
|  | |  |  |  |  |

Note: a-b= model reference to Table S2, T1=pre-treatment (baseline), T2= 3 months after baseline, T3= 6 months of baseline; T1-T2= completed material (task/videos) in % between T1 and T2 (month 0-3), T2-T3= completed material (task/videos) in % between T2 and T3 (months 3-6); *p<.05.

**Table S4.** Description of usage parameters (early and late)

|  |  | Early Use (T1T2) | | | |  | Late Use (T2T3) | | | |  |
| --- | --- | --- | --- | --- | --- | --- | --- | --- | --- | --- | --- |
| Variable |  | *M* | *SD* | min | max |  | *M* | *SD* | min | max | *p*^1^ |
|  |  |  |  |  |  |  |  |  |  |  |  |
| Login (n) |  | 5 | 4.38 | 0 | 18 |  | 0.53 | 1.20 | 0 | 9 | <.001 |
| Progress (%) |  | 31.88 | 26.08 | 0 | 96.70 |  | 1.93 | 5.83 | 0 | 31.72 | <.001 |

Note: N= 276. ^1^ Mean comparisons were made using t-tests for dependent samples.
